# Printed Textile-Based Ag_2_O–Zn Battery for Body Conformal Wearable Sensors

**DOI:** 10.3390/s21062178

**Published:** 2021-03-20

**Authors:** Akash Kota, Ashish Gogia, Amy T. Neidhard-Doll, Vamsy P. Chodavarapu

**Affiliations:** Department of Electrical and Computer Engineering, University of Dayton, Dayton, OH 45469, USA; gogiaa1@udayton.edu (A.G.); aneidharddoll1@udayton.edu (A.T.N.-D.); vchodavarapu1@udayton.edu (V.P.C.)

**Keywords:** sensor power, textile battery, stencil printing, zinc-silver oxide, battery characteristics

## Abstract

Wearable electronics are playing an important role in the health care industry. Wearable sensors are either directly attached to the body surface or embedded into worn garments. Textile-based batteries can help towards development of body conformal wearable sensors. In this letter, we demonstrate a 2D planar textile-based primary Ag_2_O–Zn battery fabricated using the stencil printing method. A synthetic polyester woven fabric is used as the textile substrate and polyethylene oxide material is used as the separator. The demonstrated battery achieves an areal capacity of 0.6 mAh/cm^2^ with an active electrode area of 0.5 cm × 1 cm.

## 1. Introduction

The rapid development of wearable devices is fueled by the interest from the general population for real-time health and wellness monitoring [1,2]. Various human health and wellness indicators including body temperature, peripheral capillary oxygen saturation (SpO_2_) level, electrocardiogram, calories burned, exercise outcomes, and walking steps can be monitored non-invasively in real time by wearable devices. Wearable devices also play an important role in the development of smart bandages for wound healing applications [3]. Unlike a conventional bandage, a smart bandage could be used to monitor the pH, biological growth factors, temperature, and moisture information at the wound location. A variety of smart bandages have been reported in the literature with the capability of measuring various wound healing parameters. For example, to monitor the pH at a wound location, different pH-sensitive polymer materials such as polyvinyl alcohol-polyacrylic acid gel and polyaniline were previously developed [4,5,6]. Apart from pH, different types of temperature, moisture, and oxygen-sensitive smart bandages have also been demonstrated [7,8,9] which can sense multiple parameters such as bleeding, pH, and pressure levels at the wound site [10]. In most reported smart bandages, the wearable sensors are powered either by a rigid battery or a radio frequency identification (RFID) tag. With the RF power harvesting technique, the RF power source or RF transmitter should be within close proximity of the bandage [11], which is a significant hurdle. On the other hand, conventional batteries are rigid, non-conformal, and add weight to the bandage. Thus, textile-based power sources are advantageous as they are thin, conformal, and light in weight.

Two different approaches have been proposed for textile-based batteries. The first approach is to fabricate a one-dimensional (1D) fiber- or yarn-shaped battery which can later be woven into a fabric. In this approach, the electrochemically active materials of the battery are loaded onto conductive textile fibers to design fiber electrodes [12]. Materials such as carbon nanotubes (CNTs) and graphene are used to design conductive fibers [13,14]. In the second approach, the electrode materials of the battery are directly loaded onto two-dimensional (2D) fabrics. Printing methods such as dip-coating and screen printing are used to design 2D textile-based batteries. Using these two approaches, different types of textile-based batteries such as lithium–ion, Ag_2_O–Zn, and MnO_2_–Zn have been demonstrated to date [15,16,17,18].

For power sources in healthcare applications such as smart bandages or wearable heart rate monitors, Ag_2_O–Zn chemistry is preferred over lithium–ion chemistry due to biosafety reasons. Furthermore, unlike lithium–ion chemistry, Ag_2_O–Zn chemistry is not water-sensitive. Therefore, it does not require an inert atmosphere during the fabrication process. In a printed battery, all the layers such as current collector, electrodes, and separator should ideally be printable. In addition, an ideal separator material in a battery should not react with its active electrode components. For alkaline battery chemistries such as Ag_2_O–Zn and MnO_2_–Zn, the separator layer should provide good conductivity for hydroxide ions where materials such as polyvinyl alcohol (PVA) and polyacrylic acid (PAA) were previously proposed to demonstrate 1D and 2D textile batteries, respectively [19,20]. Even though a PVA-based separator demonstrates high ionic conductivities, in the presence of hydroxide ions and mild oxidizing agents such as MnO_2_ and Ag_2_O, it can get oxidized to a ketone [21,22], thus limiting its usage. Unlike PVA, materials such as polyvinylidene fluoride (PVDF) and polyethylene oxide (PEO) can act as inert separator materials [23]. Using PVDF and PEO as the separator materials, MnO_2_–Zn and Ag_2_O–Zn batteries have been demonstrated on rigid substrates using flexographic and extrusion printing methods, respectively [24,25].

In this work, we demonstrate a 2D planar primary Ag_2_O–Zn battery on a flexible textile substrate which uses PEO as the separator material. A low-cost stencil printing method is used in the battery fabrication process. The process parameters of the PEO-based gel electrolyte were optimized to increase the discharge duration of the textile-based Ag_2_O–Zn battery. Specifically, we examined the effect of gel electrolyte drying time on the battery discharge capacity. Without encapsulation, the demonstrated battery achieved an areal capacity of $0.6\;\mathrm{mAh}/{\mathrm{cm}}^{2}$ with a total weight of approximately 0.460 g.

## 2. Materials and Methods

### 2.1. Materials Used

Textile Substrate: A 100% polyester fabric (Eagegof, Shenzhen, China, serial number: B07P45DHXY) was used as a textile substrate to print different components of the battery. To prevent the electrolyte wicking and to reduce the surface roughness of the polyester substrate, an insulation layer of 200 µm was stencil printed. A commercially-available insulation ink (Creative Materials, Ayer, MA, USA serial number: 127-14) was used without any modification. 

Current Collector: To print the current collector layer, carbon ink (Creative Materials, Ayer, USA serial number: 128-07) was used. The obtained carbon ink was used without any modifications. A 200-µm-thick carbon layer was stencil printed on top of the insulation layer to act as a current collector layer.

Electrodes: To prepare the electrode inks, 5 wt% PVDF (MTI, Richmond, VA, USA, serial number: 121120) binder was used. To dissolve the PVDF, N-methyl-2-pyrrolidone (NMP) (Sigma-Aldrich, Milwaukee, WI, USA, serial number: 443778) was used as the solvent. First, 10 g of 5 wt% PVDF binder was prepared by dissolving 0.5 g of PVDF in 9.5 g of NMP. The binder solution was stirred using a magnetic stirrer at 500 rpm for 12 h at room temperature. The silver oxide slurry was prepared with 5 wt% PVDF binder. The silver oxide slurry contained 95 wt% Ag_2_O (Sigma-Aldrich, Milwaukee, USA, serial number: 221163) and the rest is the binder. To prepare 1 g of 95:5 silver oxide ink, 950 mg of Ag_2_O was added to 1 g of 5 wt% PVDF binder. To prepare a homogeneous ink, the Ag_2_O slurry was mixed for 20 min in an AR-100 conditioning mixer. The zinc slurry was also prepared with 5 wt% PVDF binder. The zinc slurry contained 96 wt% Zn (Sigma-Aldrich, Milwaukee, USA, serial number: 209988), 2 wt% Bi_2_O_3_ (Sigma-Aldrich, Milwaukee, USA, serial number: 223891), and the rest was the binder. To prepare 1 g of 96:2:2 zinc ink, 960 mg of Zn and 20 mg of Bi_2_O_3_ were added to 0.4 g of 5 wt% PVDF binder. The approximate sizes of the zinc particles were <10 µm. The zinc slurry was also mixed for 20 min in an AR-100 conditioning mixer.

Separator: The separator gel was prepared by using PEO (Sigma-Aldrich, Milwaukee, USA, serial number: 182028). The mechanical strength of the separator gel plays an important role in the stencil printing process. Based on the viscosity average molecular weight $\left(M_{v}\right)$ of a polymer, its mechanical properties can be estimated. The $M_{v}$ of PEO typically varies from 12,000 to 600,000. PEO with higher $M_{v}$ exhibits good mechanical strength. Therefore, PEO having $M_{v}\sim 600,000$ was selected. A 5 wt% PEO gel was used as the separator material. To prepare 10 g of 5 wt% PEO gel, 0.5 g of PEO $\left(M_{v}\sim 600,000\right)$ was dissolved in 9.5 g of distilled water. To get a homogenized gel, the PEO solution was stirred for 4 h using a magnetic stirrer at 500 rpm at room temperature. During the stirring process, a large amount of oxygen bubbles were generated in the gel. Before stencil printing the gel, the oxygen bubbles were removed by exposing the gel to nitrogen gas for 10 min. 

Electrolyte: The 95 wt% potassium hydroxide (KOH) (Sigma-Aldrich, Milwaukee, USA, serial number: 484016) solution was used as the electrolyte. To prepare 95 wt% KOH solution, 9.5 g of KOH pellets were dissolved in 10 g of distilled water.

### 2.2. Battery Fabrication

To print different layers of the battery, we used stencils of different thicknesses. The stencil material should be selected such that it must be non-reactive to the inks that are being used. Stencils used in the printing process were made of two different materials namely, polyamide-nylon 6 (PA 6) and polyethylene terephthalate (PET). Except for the separator layer, all the remaining layers were printed using PA 6 stencil, as it has been experimentally observed that the PET stencil reacts with the electrode inks. Therefore, to print the insulation and carbon layers a 200-µm-thick PA 6 (Goodfellow, Coraopolis, PA, USA, serial number: AM301200) stencil was used. Similarly, to print the electrode layers, a 350-µm-thick PA 6 (Goodfellow, Coraopolis, USA, serial number: AM301350) stencil was used. A 500-µm-thick PET stencil was used to print the separator layer. To print the carbon and electrode layers in a rectangular shape, the stencils were engraved with required patterns using a CO_2_ laser (Boss Laser, Sanford, FL, USA, serial number: LS1416). 

**Figure 1:**
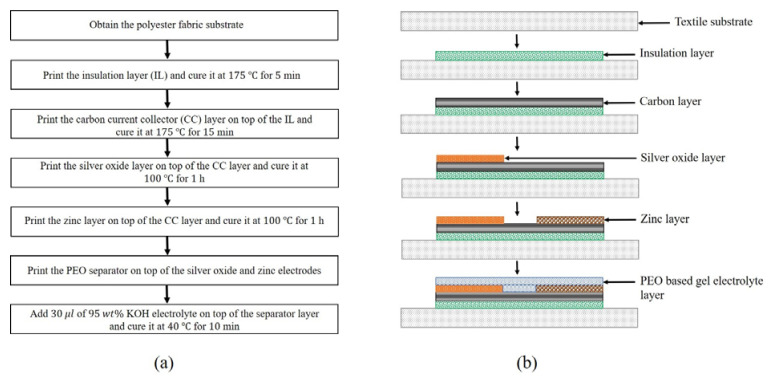
(**a**) Flow chart illustrating the battery fabrication procedure and (**b**) cross-sectional view of the corresponding printed layer at each fabrication step.

The battery printing process is as follows. First, a 100% polyester textile substrate of size 5 cm×5 cm is obtained. Using the 200-µm-thick PA 6 stencil, an insulation layer of size 1.2 cm×1.5 cm is printed on the fabric. To evaporate the solvent, the printed insulation layer is dried in a vacuum oven at 175 °C for 5 min. After drying, using the patterned 200-µm-thick PA 6 stencil, the carbon current collector layers of size 0.5 cm×1.5 cm are printed on top of the insulation layer. The spacing between the two carbon layers is 0.2 cm. The printed carbon layers are also dried in a vacuum oven at 175 °C for 15 min. After drying, the silver oxide and zinc inks must be printed on top of the carbon layers consecutively. The process to print the electrode layers is identical as above. Using the 350-µm-thick PA 6 stencil, an electrode layer of size 0.5 cm×1 cm is printed on one of the carbon layers. The printed electrode layer is dried in a vacuum oven at 100 °C for 60 min. After drying, using the 500-µm-thick PET stencil, the separator layer is printed. On top of the separator layer, using a micropipette, 30 µL of KOH is deposited. To remove the excess water in the separator layer, the battery is dried in a vacuum oven at 40 °C for 10 min. A flow chart illustrating the battery fabrication procedure is shown in Figure 1a. At each fabrication step, the cross-sectional view of the corresponding printed layer is shown in Figure 1b.

### 2.3. Theoretical Calculations

The following calculations are used to determine the theoretical capacity of the proposed textile-based Ag_2_O–Zn battery. To calculate the capacity of a typical Ag_2_O–Zn battery, it is essential to determine the weight of Ag_2_O $\left(w_{{\mathrm{Ag}}_{2}O}\right)$ present in the cell. The steps to calculate $\left(w_{{\mathrm{Ag}}_{2}O}\right)$ are explained briefly. The weight of the fabric substrate $\left(w_{f}\right)$ with printed carbon current collector layers can be written as

(1) $$w_{f}=\;xg.$$

After stencil printing the silver oxide layer on one of the carbon layers, the weight, y, is measured. Let us assume $w_{{\mathrm{Ag}}_{2}O}$, $w_{\mathrm{PVDF}}$, and $w_{\mathrm{NMP}}$ represent the weight of silver oxide, PVDF, and NMP in the silver oxide electrode, respectively. Therefore,

(2) $$w_{f}+w_{{\mathrm{Ag}}_{2}O}+w_{\mathrm{PVDF}}+w_{\mathrm{NMP}}=y.$$

From Equations (1) and (2), one can write Equation (3) as

(3) $$w_{{\mathrm{Ag}}_{2}O}+w_{\mathrm{PVDF}}+w_{\mathrm{NMP}}=z,$$

where z=y−x. Now, silver oxide electrode is dried to evaporate the NMP solvent present in it. After drying, the weight of the fabric, w, is measured. As NMP is evaporated after drying, we have only PVDF and Ag_2_O left in the silver oxide electrode. Therefore,

(4) $$w_{f}+w_{{\mathrm{Ag}}_{2}O}+w_{\mathrm{PVDF}}=w.$$

From Equations (2) and (4),

(5) $$w_{\mathrm{NMP}}=y-w.$$

By knowing the weight of NMP, the weight of PVDF can be calculated as

(6) $$w_{\mathrm{PVDF}}=0.05263\times w_{\mathrm{NMP}}.$$

From Equation (6), the multiplication factor is valid only if 5 wt% PVDF binder is used in the preparation of silver oxide ink. If the wt% of binder is different, then the multiplication factor can be calculated as

(7) $$\mathrm{wt}\%\;\mathrm{PVDF}\;\mathrm{in}\;\mathrm{NMP}=\frac{w_{\mathrm{PVDF}}}{w_{\mathrm{PVDF}}+w_{\mathrm{NMP}}}.$$

By knowing $w_{\mathrm{PVDF}}$ and $w_{\mathrm{NMP}}$, using Equation (3)

(8) $$w_{{\mathrm{Ag}}_{2}O}=z-w_{\mathrm{PVDF}}-w_{\mathrm{NMP}}.$$

The theoretical capacity (C) of the battery can be calculated as

(9) $$C=\mathrm{Specific}\;\mathrm{capacity}\;\mathrm{of}\;{\mathrm{Ag}}_{2}O\times w_{{\mathrm{Ag}}_{2}O},$$

where the specific capacity of Ag_2_O is 231.312 mAh/g. Similar calculations can be used to determine the weight of zinc $\left(w_{\mathrm{Zn}}\right)$ in the zinc electrode. Using these calculations, in a typical stencil printed battery, the weights of Ag_2_O and Zn in the electrodes were found out to be approximately 8 mg and 26 mg, respectively. 

### 2.4. Characterization Methods

The surfaces of different layers of the printed battery were characterized by using Hitachi S-4800 high-resolution scanning electron microscope (HRSEM) (Hitachi High-Tech, Dallas, TX, USA). The thickness profiles of different printed layers of the battery were obtained using Veeco Dektak 6 M surface profiler (Bruker, Billerica, MA, USA). The open-circuit voltage (OCV) and discharge profiles of the printed battery were obtained using a Neware BTS 4000 cycler (Neware Technology, Shenzhen, China). 

## 3. Results

**Figure 2:**
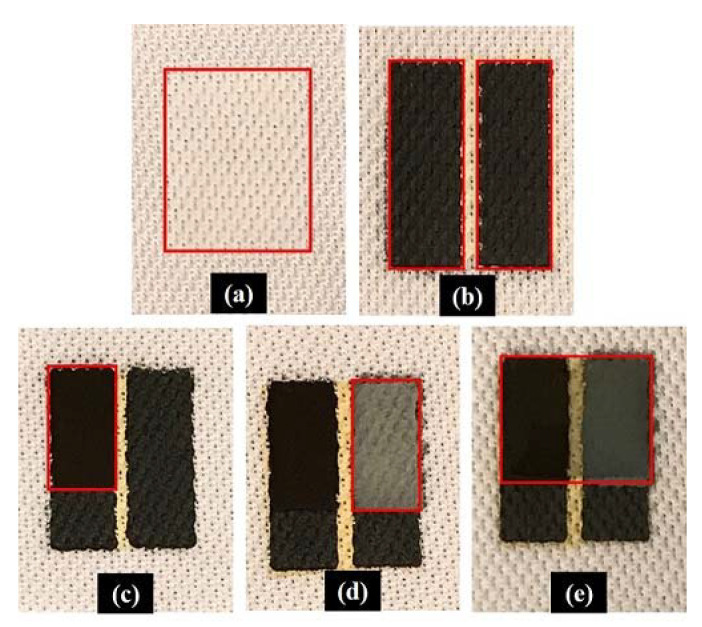
Printed (**a**) insulation layer, (**b**) carbon current collector layer, (**c**) silver oxide layer, (**d**) zinc layer, and (**e**) separator layer.

The macroscopic images of different layers of the printed battery are shown in Figure 2.

### 3.1. HRSEM Characterization

**Figure 3:**
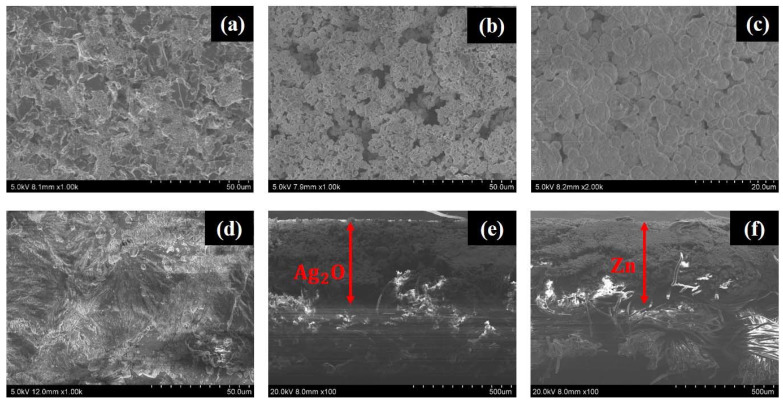
The HRSEM images of the printed layers. Top surface of the (**a**) carbon, (**b**) silver oxide, (**c**) zinc, and (**d**) polyethylene oxide (PEO)-based gel electrolyte layers. Cross-sectional SEM image of the (**e**) Ag_2_O and (**f**) Zn electrode.

The topographic HRSEM images of carbon, silver oxide, zinc, and PEO-based gel electrolyte layers of the printed battery are shown in Figure 3a–d, while the cross-sectional microscopic images of the electrodes are shown in Figure 3e,f. 

From Figure 3a it can be observed that the surface of the carbon layer is conformal, and it has no voids or micropores. The micropores present in a printed carbon layer will increase the resistance between the electrode and the current collector layer. Figure 3b,c shows the surface morphology of the silver oxide and zinc layer, showing a dense and uniformly distributed particle structure on the surface. The surface of the PEO-based gel electrolyte layer is shown in Figure 3d. The micrograph was taken after drying the gel electrolyte layer at 40 °C for 10 min. From Figure 3d it can be observed that the surface of the gel electrolyte layer is free from micropores and cracks, which ensured that no oxygen bubbles were generated during the drying process.

### 3.2. Surface Profiler Characterization 

**Figure 4:**
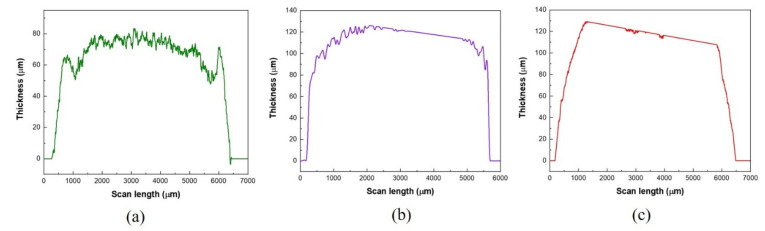
Thickness profiles of the printed (**a**) carbon current collector layer, (**b**) silver oxide layer, and (**c**) zinc layer.

The thickness profiles of the printed carbon, silver oxide, and zinc layers are shown in Figure 4. 

From Figure 4, it can be observed that the average thickness of typical carbon, silver oxide, and zinc layers are 70.67 μm, 108.68 μm, and 105.14 μm, respectively.

### 3.3. OCV and Discharge Profiles 

**Figure 5:**
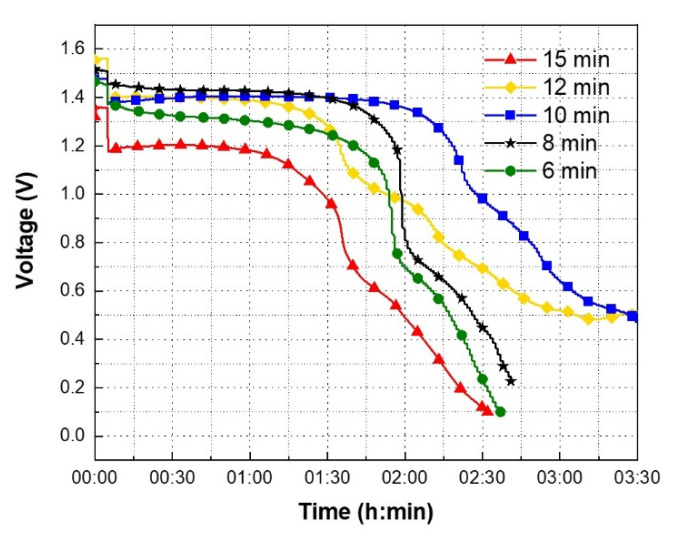
The discharge profiles of the printed batteries with 5 wt% PEO-based gel electrolyte dried at 40 °C for 6 min, 8 min, 10 min, 12 min and 15 min respectively.

The process parameters of the gel electrolyte layer, such as concentration of the electrolyte, weight percentage of the PEO, and drying conditions, play an important role in the battery discharge characteristics. Excess water present in the gel electrolyte will increase the battery’s internal resistance. To reduce the water content in the electrolyte, the concentration of KOH is kept at 95 wt%. The water content in the PEO separator can be decreased by increasing the weight percentage of PEO. However, increased weight percentage also increases the viscosity of the final PEO gel, which is not stencil printable. From the experiments it was observed that a 5 wt% PEO gel is able to give a conformal coating on the planar printed electrodes. However, the printed separator layer still had 95 wt% water, which confirmed that a drying step was inevitable. After depositing the electrolyte on the printed separator, the batteries were dried at 40 °C in different time intervals. As the Ag_2_O–Zn battery is sensitive to heat, it is essential to determine the optimum drying time. The over- and under-drying of the gel electrolyte layer results in the decreased performance of the battery. The optimum drying time for the gel electrolyte is determined by observing the discharge profiles. Figure 5 shows the discharge profiles of the printed batteries with 5 wt% PEO-based gel electrolyte dried under vacuum at a constant temperature of 40 °C for 6 min, 8 min, 10 min, 12 min, and 15 min, respectively.

In Figure 5, between 0.1 h and 2.25 h, whichever discharge profile has the smallest variation in the voltage, the drying time associated with the corresponding discharge profile is considered as the optimum drying time. After performing the drying step, all the printed batteries were discharged under a constant current load condition of 100 µA. Before discharging, all the batteries were rested for 5 min to record the OCV. The OCV and discharge voltages of the batteries at different time instances are summarized in Table 1. 

**Figure 6:**
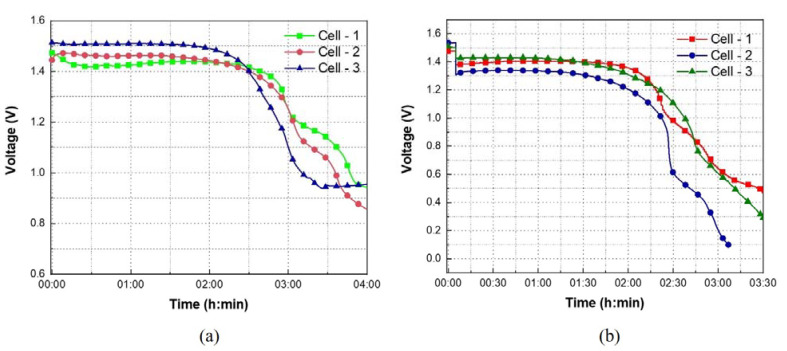
The (**a**) OCV and (**b**) discharge profiles of the stencil printed batteries with 5 wt% PEO-based gel electrolyte dried at 40 °C for 10 min, repeated in triplicate.

In Table 1, the variable V1 represents the OCV of the battery. From Figure 5 it can be observed that all the batteries were in the rest cycle for the first 5 min. During the rest cycle, the OCV of the batteries which were dried for 6 min, 8 min, 10 min, 12 min, and 15 min are illustrated in Table 1. After 5 min, all the batteries enter discharge mode. The variables V2 and V3 in Table 1 represent the voltage of the battery at 6 min and 135 min, or 2.25 h, respectively. The variable ΔV represents the percentage difference between V2 and V3. In Figure 5, for the battery which was dried for 10 min, between 0.1 h and 2.25 h, the battery voltage is varied between 1.39 V to 1.18 V. Similarly, for the batteries which were dried for 6 min, 8 min, 12 min, and 15 min, between 0.1 h and 2.25 h, the battery voltage is varied between 1.39 V to 0.44 V, 1.47 V to 0.58 V, 1.42 V to 0.75 V, and 1.24 V to 0.22 V, respectively. For the batteries which were dried for 6 min, 8 min, 10 min, 12 min, and 15 min, between 0.1 h and 2.25 h, the battery voltage is decreased by 68.35%, 60.54%, 15.11%, 47.18%, and 82.26%, respectively. The variation in the battery voltage is minimum when the gel electrolyte layer was dried for 10 min. Based on the data shown in Table 1, the drying times which were below and above 10 min can be considered as under- and over-drying times. When the battery was dried for less than 10 min, the excess water present in the gel electrolyte was not completely evaporated. From Table 1, when the drying time is increased from 6 min to 10 min, ΔV is decreased from 68.35% to 15.11%. This confirms that the gel electrolyte layer has appropriate amount of moisture when it is dried at 10 min. When the drying time is further increased from 10 min to 15 min, the gel electrolyte is over-dried, thus increasing the ΔV from 15.11% to 82.26%. Therefore, it can be concluded that the optimum drying time for the gel electrolyte is 10 min. To demonstrate repeatability, multiple batteries were tested to obtain the OCV and discharge profiles. The OCV and discharge profiles of the printed batteries with 5 wt% PEO-based gel electrolyte dried at 40 °C for 10 min are shown in Figure 6.

In Figure 6a, the OCV profiles of the printed batteries are shown for 4 h. For instance, in the OCV profile of cell-2 between 0 to 2 h, the OCV of the battery is varied between 1.4735 V to 1.4369 V. From 2 to 3 h, OCV is dropped from 1.4369 V to 1.2493 V. Similarly, between 3 to 4 h, the voltage is further dropped from 1.2493 V to 0.9421 V. Between 0 to 2 h and 2 to 4 h, the OCV is decreased by 2.48% and 34.44%, respectively. In Figure 6b, the discharge profiles of the printed batteries are shown for 3.5 h. Before discharging, the battery was rested for 5 min to observe the OCV. For instance, in Figure 6b, during the rest cycle, the OCV of cells 1, 2, and 3 is 1.47 V, 1.53 V, and 1.51 V, respectively. After 5 min of rest cycle, the batteries were discharged under a constant current load condition of 100 µA. In Figure 6b, for cell-1 during the discharge process, between 0.083 to 2 h, the battery voltage is varied between 1.3932 V to 1.3373 V. From 2 to 2.75 h, the voltage is dropped from 1.3373 V to 0.7871 V. Between 0.083 to 2 h and 2 to 2.75 h, the battery voltage is decreased by 4.01% and 41.14%, respectively. After 3.5 h, all the three cells achieved a discharge capacity of 0.3 mAh. 

In addition, from Figure 6a it can be observed that the OCV profile of cells 1, 2, and 3 are not the same. Similarly, in Figure 6b, there is a variation in the discharge profiles. The slight variations in the OCV and discharge profiles could be attributed to the process variations under laboratory conditions. In all samples, while the corresponding Ag_2_O, Zn, and PEO layers in all three consecutively printed batteries is approximately the same, the distribution of electrolyte in their respective separator layers might not be uniform and will be optimized in our future research. As the KOH electrolyte is deposited manually using a micropipette, the electrolyte diffusion in the separator layer is not uniform, thus, showing the variation in OCV and discharge profiles. As the OCV and discharge measurements of the batteries were performed at a room temperature of 22 °C, throughout the experiment, it is expected that the electrolyte dehydration phenomena took place. This phenomenon can be avoided by printing an encapsulation layer on top of the PEO gel electrolyte layer. However, even without the encapsulation layer, the printed battery can provide a stable current of 100 µA with a voltage of 1.3 V for up to 2 h, achieving an areal capacity of $0.6\;\mathrm{mAh}/{\mathrm{cm}}^{2}$. In Table 2, the achieved areal capacity is compared with other alkaline batteries which are demonstrated on flexible substrates. 

In Table 2, the reported areal capacity of the stencil printed primary MnO_2_–Zn battery which used PVA/cellulose as the separator material is $0.8\;\mathrm{mAh}/{\mathrm{cm}}^{2}$ [26]. Similarly, the reported aerial capacity of Ag_2_O–Zn battery which uses SIS as the binder material and PAA as the separator material is 2.5 mAh/cm^2^ [20]. As the demonstrated battery is not encapsulated, the achieved aerial capacity is less compared with that of the other alkaline batteries. However, the achieved aerial capacity is comparable with that of MnO_2_–Zn battery which used PVA/cellulose as the separator material. The areal capacity of the demonstrated textile battery can be further increased by encapsulating it properly. The encapsulation materials must be inert to alkali solutions, and at the same time, they must have good adhesion properties with textile substrates. Materials such as thermoplastic polyurethane (TPU) films are used as encapsulation materials for printed interconnect layers on textile substrates to increase their durability [27]. The TPU film is composed of two layers: a polymer film layer and an adhesive layer. The TPU films are adhered to the textile substrates by heat lamination. Even though TPU films exhibit strong adhesion properties, the inertness of the TPU films towards concentrated alkali solutions must be established for their usage in the design of textile-based batteries. Proper encapsulation of the battery will prevent the dehydration of the PEO-based gel electrolyte, thereby enhancing the aerial capacity. In real-world use, it also prevents the electrolyte contact with the skin, thus ensuring the safe operation of the battery.

## 4. Conclusions

In this work, a low-cost stencil printing method was used to demonstrate a textile-based primary Ag_2_O–Zn battery which used PEO as the separator material. All the layers of the battery were processed on a textile substrate. The 5 wt% PEO gel acted as a stable separator material without showing any sign of delamination after adding the KOH electrolyte. The drying time of the PEO-based gel electrolyte was optimized to increase the discharge duration of the textile-based Ag_2_O–Zn battery. The optimum drying time for the 5 wt% PEO-based gel electrolyte was found to be 10 min at 40 °C. The demonstrated battery achieved an areal capacity of 0.6 mAh/cm^2^. Proper encapsulation materials suitable for the textile substrates are needed to improve the areal capacity further.

## Figures and Tables

**Table 1:** The open-circuit voltage (OCV) and discharge voltages of the batteries which were dried at 6 min, 8 min, 10 min, 12 min, and 15 min, respectively.

Drying Time	OCV (V1)	Voltage at 6 min (V2)	Voltage at 135 min (V3)	ΔV (%)
6 min	1.45 V	1.39 V	0.44 V	68.35%
8 min	1.51 V	1.47 V	0.58 V	60.54%
10 min	1.47 V	1.39 V	1.18 V	15.11%
12 min	1.56 V	1. 42 V	0.75 V	47.18%
15 min	1.35 V	1.24 V	0.22 V	82.26%

**Table 2:** Aerial capacity comparison with other alkaline batteries.

Battery	Separator Material	Binder Material	Printing Method	Aerial Capacity (mAh/cm^2^)
Ag_2_O-Zn	PEO	PVDF	Stencil printing	0.6 [This work]
MnO_2_-Zn	PVA/Cellulose	Styrene-butadiene rubber	Stencil printing	0.8 [26]
Ag_2_O-Zn	PAA	Polystyrene-*block*-polyisoprene-*block*-polystyrene (SIS)	Screen printing	2.5 [20]

## Data Availability

Research data presented in this article are available on request from the corresponding author.
